# Digital inclusion: A mixed-method study of user behavior and content on Twitter

**DOI:** 10.1177/20552076231211277

**Published:** 2023-11-01

**Authors:** Martin Salzmann-Erikson

**Affiliations:** 1 Faculty of Health and Occupational Studies, Department of Caring Sciences, University of Gävle, Gävle, Sweden

**Keywords:** digital exclusion, digital inclusion, digitalization, information and communication technology, literacy, informatics, social media

## Abstract

**Objective:**

This study is the first to explore user behavior and characterize the content shared about digital inclusion on Twitter.

**Methods:**

This mixed-methods research consists of 14,000 tweets featuring the hashtag “#digitalinclusion,” posted on Twitter over 15 months. A machine learning technique, latent Dirichlet allocation, was utilized to discover abstract topics within the tweets statistically. The algorithm identified important keywords and text associated with each topic by modeling the underlying word co-occurrence patterns in the dataset. A manual qualitative content analysis was applied to the qualitative data (1000 tweets).

**Results:**

Tweets containing #digitalinclusion are driven by four motives: 1) warning against the risks of digital exclusion; 2) tweets that promote actions to increase digital inclusion; 3) tweets that call for others to take action to improve digitalization; and 4) tweets that are neutral but fuel the debate by being active. Quantitative analysis revealed that users discussing digital inclusion come from various continents, including the USA, Europe, Africa, and Asia. There were 3931 unique user accounts, with individuals posting between one and 368 tweets. Approximately half of the tweets contained some embedded media.

**Conclusion:**

The study concludes that digital inclusion is a subject that engages Twitter users worldwide. Tweets that were associated with community and local initiatives and sustainable development had the highest engagement in terms of the number of retweets and likes. The interpretation is that digital inclusion is crucial for achieving equity in living conditions and enhancing access to health information and services. While initiatives to increase digital inclusion are underway, Twitter users call for more efforts to prevent growing digital exclusion. Twitter, as a social media platform, is valuable for studying the motivations that drive digital inclusion and help counter digital exclusion.

## Introduction

Digital inclusion has been described as being of vital importance because it can help to reduce disparities in access to information and increase opportunities to promote social and economic development.^1,2^ Digital inclusion is defined as “the activities necessary to ensure that all individuals and communities, including the most disadvantaged, have access to and use of Information and Communication Technologies (ICTs).”^3^ Several of the Sustainable Development Goals (SDGs) in Agenda 2030 are highly intertwined with the ambition of increasing digital inclusion, for example SDG 3: Good Health and Well-being; SDG 4: Quality Education; and SDG 10: Reduced Inequalities.^4^ Initiatives are taken globally, but research points to the existence of digital divides and digital equities between groups of people living in cities and those living in rural environments.^5,6^ Digital inclusion plays a pivotal role for thriving among individuals, communities, and societies around the world. In an editorial paper, Reisdorf and Rhinesmith^7^ argued for the relationship between digital inclusion and social inclusion. They stressed the importance of transforming ideas about “‘what can be done’ rather than focusing on ‘what is amiss’” (p. 135). Hence, ensuring digital inclusion presupposes collaborative initiatives taken by governments, businesses, and civil society organizations. Calls for digital inclusion are highly evident in governmental policy documents and discussed in the academic literature. However, little research has been carried out on how digital inclusion is discussed in digital venues. Thus, this is the first research article to explore and describe how Twitter users behave when sharing information about digital inclusion.

### Digital inclusion presupposes access to technology

According to an overview,^8^ there were about 5.48 billion unique mobile phones in the world, which is 69% of the total number of people on a global scale. About 63% of the global population use the web, and 59.3% of the population are active social media users. Despite the rapid increase in individuals who use the web and have access to mobile phones, the numbers can also be read as indicating that 36.5% of the world's population do not use the web; these people are, thus, on the outskirts of digitalization and risk becoming digitally excluded.

Digital inclusion refers to having access to the web, access to digital devices, and access to digital support, all of which help people to develop the skills needed to use technologies effectively.^2^ It has been emphasized that ensuring that individuals have access to the web is a crucial part of digital inclusion.^9^ This lack of adequate broadband infrastructure impacts both rural and urban communities, for example 38% of households in the Bronx in New York do not have broadband at home.^10^ Access is not only restricted to infrastructure, but is also a matter of individual capacity. It has been stressed that threats to digital inclusion also involve impaired individuals who face physical or cognitive challenges that make it difficult for them to use technology.^11,12^ Ensuring that individuals and communities have access to these services can help to improve their quality of life and provide new opportunities for social and economic development. Two indicators that positively correlate with digital inclusion are having a computer and having an internet connection at home.^13^

### Facilitators and barriers to fuel digital inclusion

Facilitators and barriers are things which motivates behavior. In this study, tweets are representations of acts, attitudes, or conceptualizations of what drives or obstructs digital inclusion: facilitators and barriers. Below are examples of a few of the initiatives taken to fuel digital inclusion. One long-term project in Chile increased schoolchildren's connectiveness to the internet.^14^ Participants engaged in LinkedIn Learning Paths and provided feedback on their experiences. A significant 80% reported acquiring a new skill, while 91% successfully completed their selected path. The positive reception of the workshops highlights their potential in enhancing digital skills and fostering workforce advancement.^15^ These are all examples of concrete actions taken that are in line with the SDG in Agenda 2030.^16^ Facilitators and barriers are also a matter of changing attitudes toward ICT. This was made explicit in a qualitative study from South Africa on women's use of ICT, in which one respondent expressed a view that computers are for businesses and schools.^17^ Thus, digital inclusion is not merely about access and infrastructure, but also a matter of educating individuals to use ICT.

### Digital literacy – and nursing informatics

Digital literacy refers to individuals’ abilities to use ICT effectively and appropriately as well as to the skills needed to find, evaluate and use information from a variety of sources. Thus, digital literacy plays a vital role in digital inclusion. Individuals who have not had opportunities to receive an education lack the knowledge and skills needed to use technology, thus they have low digital literacy and risk being marginalized and digitally excluded.^18–20^ Digital literacy training can help to bridge the gap between those who are already familiar with ICT and those who are not.^21–24^ One study of the long-term effects of digital literacy programs showed that people not only gain confidence in using technology, but also experience increased empowerment and sense of self-efficacy.^25^ Also, a study from the UK reported that use of digital technologies among people with social disadvantages was low and that these people did not make use of the opportunities internet could have provided them, even though internet access itself was not an issue.^26^

Digital literacy is a prerequisite for taking advantage of available health informatics. From health informatics systems, service users and patients can access e-health services as well as use electronic health records and other health information systems. Electronic health records allow service users and patients to access their health information and communicate with their healthcare providers electronically, which can improve the efficiency and effectiveness of healthcare delivery. For example, patients may be able to view their medical history, test results, and treatment plans online, and they may also be able to communicate with their healthcare providers through secure messaging or video visits. Thus, from the perspective of nursing, digital inclusion is of the utmost importance in ensuring that all patients can benefit from and thrive using digitalization, but improving patients’ empowerment and self-efficacy in digital literacy is also important.^27,28^

### Social media and societal changes

According to recently published data, among users in the age range 16 to 64 years, the average daily spent time on social media was 148 min.^8^ Using social media is far from limited to liking and sharing “cute cat” content. Rather, social media have offered powerful tools for facilitating political activism and pushing for gender equity. Social media have made it easier for individuals and organizations to mobilize, coordinate, plan, and execute collective actions, such as protests, campaigns, and other forms of activism.^29,30^ For example, social media have been used to organize protests and to raise awareness about women's right regarding sexual assault and harassment. Even though this movement began already in 2006 prompted by Tarana Burke, it first gained rapid and global attention in October 2017 in the form of #MeToo.^31^ Another well-known example for human rights, the Arab Spring, received widespread recognition in December 2010. Another large-scale project in India, the National Digital Literacy Mission, aimed to put India in a leading position in the area of digital inclusion. One particular part of that program was to empower women to use ICT.^32–34^ However, programs and initiatives do not need to be nationwide, large-scale and long-term efforts. In contrast, a small-scale initiative was reported from South Africa in which librarians helped homeless people to increase their digital literacy.^35^ A last example was the El Paso Health Education and Awareness Team at Texas Tech University Health Sciences Center organized technology education workshops in both English and Spanish to address the social determinants of health in underserved communities. Moreover, less well-known campaigns have been reported on in articles. To mention a few examples, Ali^36^ reported about Sudanese women's disobedience on Facebook in her netnographic study, and parent-led activism to acknowledge the rights of their disabled children was discussed by Carey et al..^37^ As demonstrated in these examples, if individuals are given a platform on which to share and discuss their ideas, experiences, and perspectives with a large and diverse audience, it is possible to make changes that affect local or global injustices. These online communities can be powerful forces, as they can provide individuals with a sense of belonging, solidarity, and empowerment. Thus, social media have been shown to raise awareness about important issues and spark public debate and dialogue; they can also play a significant role in driving societal changes. However, social media platforms also provide avenues for the spread of misinformation, hate, and abusive behavior online.^38^

### Twitter – a place for discussions

Users of Twitter can post messages, which are called “tweets.” Tweets are limited to 280 characters or fewer and can include text, hashtags, links, and images. Users can create tweets by logging into their Twitter account and clicking the “Tweet” button, or by using a third-party application that is connected to their Twitter account. Tweets can be seen by other users who follow the user who created the tweet, and they can also be viewed by anyone who searches for the tweet or clicks on a link to the tweet. To increase the visibility of a tweet, one or several hashtags can be used, which allows others to identify all tweets that have been posted on a specific hashtag. Tweets can be liked, retweeted (reposted by another user), and replied to by other users. Tweets are often used to share news, updates, opinions, and personal thoughts with a wide audience. They are also used to engage with other users, participate in discussions, and join online communities. Tweets can be about any topic and can be created by individuals, organizations, and businesses. On Twitter, a reply is a type of message that is directed at a specific user or group of users and is meant to respond to a previous tweet. To create a reply, a user can click the “Reply” button underneath a tweet or use the “@” symbol followed by the username of the person they are replying to. A quote is a type of tweet that allows a user to repost another user's tweet and add their own commentary or thoughts. To create a quote, a user can click the “Quote Tweet” button underneath a tweet and then add their own message in the text field provided. The original tweet will be displayed as a quote, with the username of the original author and the date of the tweet included.

Twitter has been utilized to mine public opinions and attitudes in previous studies. For example, a sentiment analysis of tweets related to COVID-19 lockdown policies in Indonesia, Suratnoaji, et al.^39^ found the public held a range of perspectives, with 14.8% expressing positive sentiment, 17.5% negative sentiment, and 67.7% neutral or uncategorized sentiment regarding the lockdowns. Another study by Bacsu et al.^40^ used thematic analysis of Alzheimer's disease-related tweets and identified four main themes including lack of awareness, social isolation, caregiver burden, and empathy. These studies demonstrate how analyzing Twitter discourse can provide insights into public opinions and attitudes on health and social issues.

### Rationale and purpose

The scientific study of digital inclusion is a significant and an expanding field that has the potential to shape policies and practices that can reduce digital inequality and foster social and economic development. As such, the importance of digital inclusion and the benefits it brings cannot be underestimated given the current state of global inequity. The scientific study of digital inclusion often involves examining the factors that contribute to digital inequality and evaluating the effectiveness of interventions designed to promote digital inclusion. From the perspective of nursing, health and digital literacy are vital to service users and patients who wish to access health information and communicate with their healthcare providers electronically. Previous research has employed both quantitative and qualitative methods, such as surveys, interviews, and case studies. However, no previous studies have analyzed how digital inclusion is discussed on social media. The present study aims to examine user behavior and describe the content shared regarding digital inclusion on Twitter.

## Material and methods

This study employs a retrospective and cross-sectional observational research design. Data were obtained from Twitter (www.twitter.com) using Export Data (www.exportdata.io). Inclusion criteria required that tweets contain the hashtag “#digitalinclusion.” The hashtag #digitalinclusion was specifically chosen as the sole focus for this study to keep the conceptual focus narrow and avoid conflating digital inclusion with related but distinct concepts that may be represented by other hashtags. An initial review of potentially relevant hashtags was conducted, but ultimately #digitalinclusion was selected to maintain a tight topical focus on tweets discussing digital inclusion itself rather than related issues like digital equity or digital literacy that may dilute the analysis. No specific exclusion criteria were established.

### Qualitative analysis

A sample of the most recent 1000 tweets was collected with the objective of conducting a qualitative exploratory examination of the themes evident in the data. The rationale for this initial sample size was twofold: first, the qualitative analysis aimed to provide an exploratory, subjective description of the themes identified in the tweets; second, the qualitative component was not designed for generalizability or for quantifying theme prevalence but rather to offer a subjective description.^41,42^ To ensure the trustworthiness of the qualitative aspect of the study, we adhered to the criteria outlined by Lincoln and Guba,^43^ which include credibility, transferability, dependability, and confirmability. These criteria have also been reiterated by Nowell et al.^44^ specifically for thematic analyses. specified for thematic analyses.

The tweets were published between October 24, 2022, and November 30, 2022. For the non-numerical data analysis, a thematic approach was employed.^41^ Initially, all tweets were read to gain a comprehensive understanding of the data, contributing to the study's credibility. Subsequently, individual tweets were coded with labels such as “getting involved with digitalization,” “story of digital inclusion,” and “improvements.” These codes were reviewed and revised as necessary before being compared for similarities and differences, thereby enhancing the study's dependability. Due to the qualitative nature of this research, neither the frequency nor the predominance of codes within each theme was calculated or reported. A visual representation of the qualitative process is provided in Table 1, contributing to the study's confirmability. During the qualitative analysis, quantitative information regarding user behavior emerged as an area warranting further investigation. As a result, the understanding of digital inclusion was further augmented with a quantitative analysis. Although the study is not intended for generalizability, thick descriptions of the data, including quoted tweets and contextual information, have been provided to allow for case-to-case transfer, thereby addressing the criterion of transferability.^43,44^

**Table 1.** Example inductive coding in the qualitative analysis.

Tweet	Code	Tentative theme	Defined themes
*We will be carrying out practicum sessions in Kisumu this week, to help our community leaders improve their facilitation skills as they impact youth in their communities. Keep checking our pages for more updates! #digitalinclusion #youthleadership #communityleaders*	We have carrying out	Our accomplishments	Upvoting and broadcasting initiatives and efforts to improve digital inclusion
*Digital inclusion should consider both your employees and customers to ensure they have access to digital tools and the skills and training required. Read more here -* https://t.co/OiKQwb1xeG *#digitalinclusion #DigitalDivide #DigitalEquality*	Digital inclusion concerns several parties who have rights to digital access	Improve skills and knowledge	Fueling the debate
*Off-campus students lack equitable access to #broadband, researchers say* https://t.co/t9E6bzvvgl *via @HigherEdDive #DigitalInclusion #DigitalEquity #highereducation* https://t.co/Nxj6q6k6zT	Off-campus students lack equitable access to broadband	Criticism and digital divides	Criticizing the discourse and warning about digital exclusion
*A lack of digital skills and access can have a huge negative impact on a person's life, leading to poorer health outcomes and a lower life expectancy, increased loneliness and social isolation, less access to jobs and education.* https://t.co/XqWxGuq8W3 *#digitalinclusion* https://t.co/fAa6HpfnQz	Lack of digital skills and access can have a huge negative impact on a person's life and digital exclusion	Criticism and digital divides	Criticizing the discourse and warning about digital exclusion

### Quantitative analysis

To accommodate this expanded focus, the initial aim was broadened to include the examination of user behavior alongside the description of content shared on Twitter regarding digital inclusion. Given that 1000 tweets were deemed an insufficient sample size for a robust quantitative analysis, the time range was extended to encompass fifteen months, from September 22, 2021, to December 29, 2022. This resulted in the inclusion of an additional 13,000 tweets from Export Data for quantitative analysis. The original 1000 tweets used for qualitative analysis were also included in the quantitative data set for a total of 14,000 tweets. This timeframe was selected to capture a snapshot of the ongoing debate on digital inclusion rather than to study long-term trends. Quantitative data were analyzed using descriptive statistics and are presented in the Results section as frequency counts, means, medians, and percentages.

#### Latent dirichlet allocation (LDA)

The analysis of the dataset, which consisted of tweets containing the hashtag “#digitalinclusion,” was conducted using LDA a generative probabilistic model for collections of discrete data such as text corpora. The LDA model is a three-level hierarchical Bayesian model, where each item of a collection is modeled as a finite mixture over an underlying set of topics. The method has shown to be effective in identification of topics in large data sets, especially based on Twitter data.^45^ Each topic is, in turn, modeled as an infinite mixture over an underlying set of topic probabilities. The first step in the process was data preprocessing. This involved tokenization, where each tweet was broken down into individual words or tokens. This was followed by the removal of common English stop words, such as “the,” “and,” “is,” etc., which do not contribute to topic modeling. Additionally, words that appeared less than 15 times in the entire corpus and words that were present in more than 50% of the documents were also excluded. This was done to ensure that the words used in the analysis were meaningful and contributed to the understanding of the topics. Following preprocessing, the tweets were transformed into a bag-of-words representation. This is a representation of text that describes the occurrence of words within a document. It involves two things: a vocabulary of known words and a measure of the presence of known words. It is called a “bag” of words because any information about the order or structure of words in the document is discarded. The model is only concerned with whether known words occur in the document, not where in the document. Subsequently, the LDA model was applied to the bag-of-words matrix. The LDA model assumes that each document in the corpus is a mixture of a small number of topics and that each word's creation is attributable to one of the document's topics. LDA then tries to backtrack from the documents to find a set of topics that are likely to have generated the collection. The LDA model was implemented using the Gensim (version 4.3.1) Python library with standard hyperparameters. In addition, the LDA relied on the libraries NumPy (version 1.23.5) and SciPy (version (1.9.1), as these provide the underlying mathematical operations used by Gensim. The number of topics was set to 10, based on evaluating the model's coherence score, which measures the degree of semantic similarity between high scoring words in each topic. This ensures the topics generated are coherent and meaningful. Once the LDA model was trained on the tweet corpus, it was used to assign each tweet to the topic that had the highest probability given the words in that tweet. This resulted in each tweet being assigned to one of the 10 topics learned by the model. The top 10 keywords for each topic were then extracted based on the words that had the highest probability of being generated by that topic. For example, the top words for Topic 0 included “tech,” “digit,” “technolog,” “peopl,” “work,” “huawei,” “reason,” “support,” “trust,” “onlin” (see Table 2). By examining these keywords, this topic was interpreted as relating to the use of technology and digital tools in people's work and online activities. Finally, to gain additional insight, the relationship between the LDA topics and user engagement metrics like retweets and likes was analyzed. The tweets were grouped by their assigned topic, and the average number of retweets and likes per topic was calculated. Linear regression, utilizing the sklearn library (version 1.1.2), was used to analyze which topics were most likely to be retweeted and liked, providing a quantitative indicator of the topics that resonated most strongly with the Twitter audience.

**Table 2.** LDA modeling topics and interpretive description.

Topic #	Stemmed words reduced to their root form from “Tweet Text”	Interpretive description of the topic content.
0	*tech, digit, technolog, peopl, work, huawei, reason, support, trust, onlin*	Discussions in this sphere revolve around the intersection of technology, digital tools, and people's interaction with them in their work or daily lives. It underscores the role of tech companies like Huawei in advancing digital inclusion and addresses trust issues related to technology and online platforms.
1	*digit, access, educ, technolog, inclus, employ, peopl, women, health, mentalhealth*	The focus here is on the digital divide and its impact on education, particularly during the COVID-19 pandemic. The conversation emphasizes the importance of internet access and digital literacy in ensuring equitable education opportunities.
2	*digit, inclus, access, help, support, onlin, skill, need, peopl, devic*	Conversations under this topic delve into digital inclusion in the context of government policy and public services. The discourse emphasizes the transformative role of digital technology in enhancing civic engagement and public service delivery.
3	*digit, communiti, work, great, thank, free, skill, connect, learn, week*	The spotlight in this topic is on the role of digital technology in business and economic growth. The narrative discusses how digital inclusion can drive innovation, entrepreneurship, and economic development.
4	*digit, inclus, access, support, learn, digitalequ, communiti, work, thank, project*	Centered around social justice and equality, this topic highlights the importance of digital access and literacy in promoting social inclusion and reducing inequalities.
5	*connect, digit, help, digitalequ, broadband, work, support, peopl, communiti, internet*	The healthcare sector takes center stage in this topic. The discussions revolve around how digital inclusion can enhance healthcare access and outcomes, particularly in the context of the COVID-19 pandemic.
6	*digit, today, access, peopl, inclus, event, join, support, thank, work*	Community development and local initiatives are the focus here. The discourse emphasizes the role of digital technology in fostering community engagement and local development.
7	*work, peopl, join, digit, look, come, session, support, great, onlin*	With a focus on environmental sustainability, this topic discusses how digital inclusion can contribute to climate action and sustainable development.
8	*digit, help, learn, inclus, access, skill, book, digitalequ, want, free*	In this topic, the discussions center around digital inclusion in the context of global development and international cooperation. The narrative highlights the role of digital technology in promoting global connectivity and cooperation.
9	*access, digit, inclus, digital, aaatraq, websit, learn, africa, year, digitalid*	The role of digital technology in media and communication is the focus here. The conversation discusses how digital inclusion can enhance media access and diversity, and foster open and inclusive communication.

## Results

For the current mixed-methods study, the results from the quantitative data analysis are first presented under several headings (3.1.1–3.1.4), and then the qualitative results are presented in themes under additional headings (3.2.1–3.2.4). Individuals, their names and/or account names are blurred, but not organizational accounts. The annual registration of Twitter accounts is visualized in Table 2.

### Characteristics of tweets

The characteristics of tweets were measured using length, retweets, likes, languages, hashtags, mentions, media type, media inclusion, emojis, and URLs. Of the 14,000 tweets, 16.9% (n = 2359) were characterized as *Quotes*, 9.2% (n = 1287) were *Replies*, and the remaining 10,352 were original *Tweets* (74.0%). In the forthcoming results, these are not treated as sub-samples. Of all tweets, 9.2% (n = 1285) originated from a verified account on Twitter.

#### Retweets and likes

The number of characters used in the tweets ranged from 17 to 491. The median length was 237 and the mean was 217.7, SD = 63. Retweets ranged between 0 and 179 (mean 1.62; median 1; SD = 3,35). 6426 tweets (45.9%) were never retweeted, 21.6% (n = 3017) were retweeted once, 12.1% (n = 1691) were retweeted twice, and 1015 tweets were retweeted three times (7.25%). Only 268 (1.9%) of the tweets were retweeted ten times or more.

The most liked tweet was liked 278 times. 675 tweets (4.8%) were liked fifteen times or more. 5.7% (n = 804) of the total tweets were liked 10–14 times. 3291 tweets (23.5%) were not given any likes at all, and 2707 tweets (19.3%) were like once. 94.5% (n = 13233) were classified as English. On the sub-sample of non-English tweets (n = 767), the most frequent language, Italian, amounted to 12.7% (n = 97) of the tweets. The LDA revealed that tweets belonging to Topic 7 have the highest average number of retweets, while tweets belonging to Topic 6 have the highest average number of likes. This suggests that these topics may be more engaging or popular among the audience. Also see Table 3 for the average number of retweeets and likes for each dominant topic.

**Table 3.** Average number of retweets and likes for each dominant topic identified by the latent dirichlet allocation (LDA) model.

Dominant Topic	Average Retweets	Average Likes
0	1.52224	4.40569
1	1.73417	4.34671
2	1.48476	3.28012
3	1.74804	4.70307
4	1.64698	4.47425
5	1.6768	4.04734
6	1.73265	5.26323
7	1.91888	5.12231
8	1.49747	3.49662
9	0.934827	2.31263

#### Hashtags and emojis

**Figure 1.**
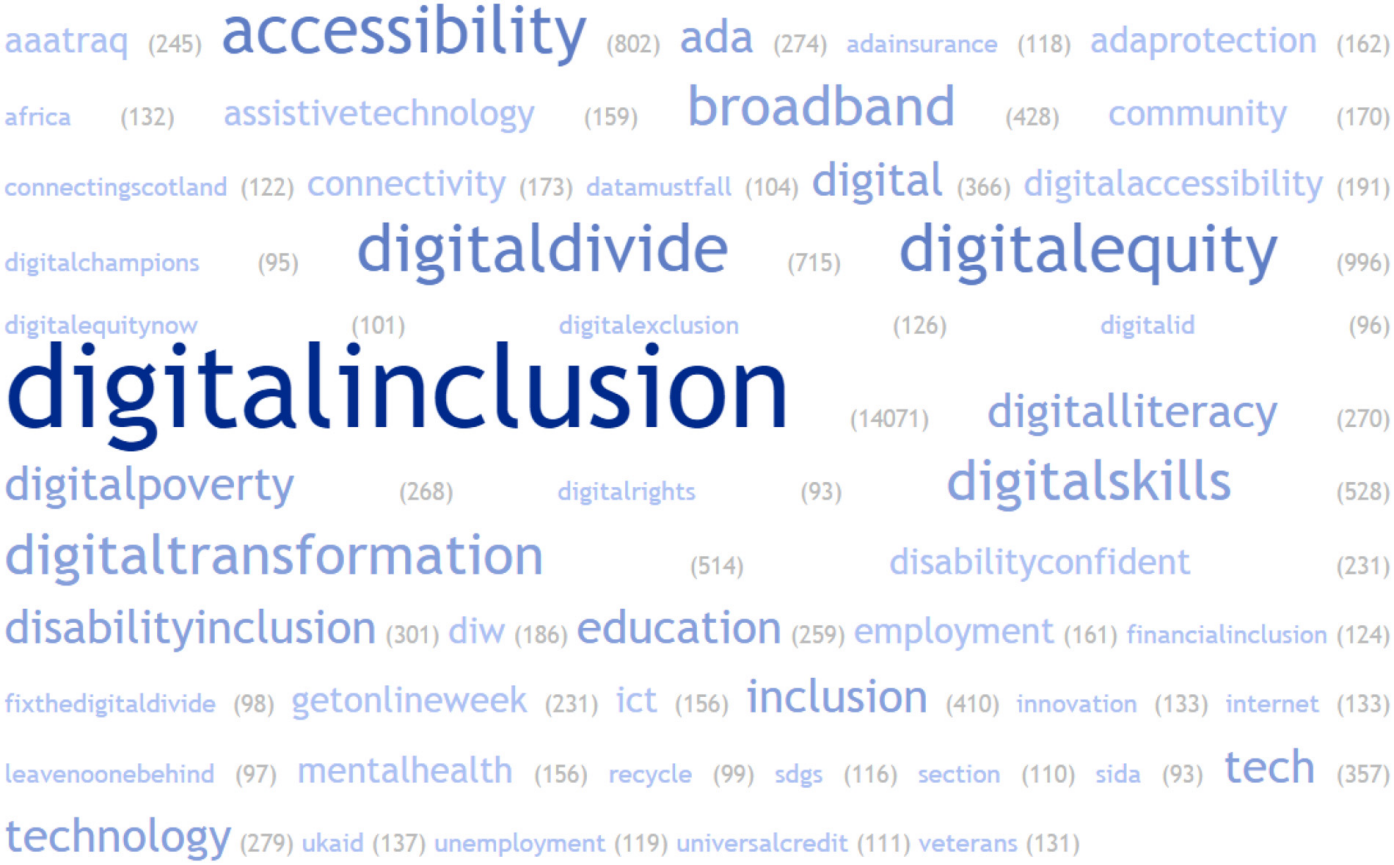
Word cloud of the hashtags in the total dataset.

**Figure 2. fig2-20552076231211277:**
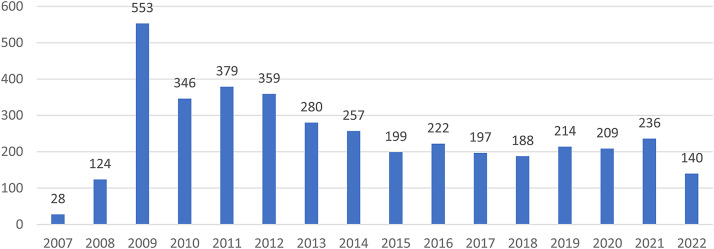
Twitter account registered yearly.

The mean number of hashtags used in tweets was 3.5 with a median of 3, ranging from one to 27. 2090 (14.9%) of the tweets used “#digitalinclusion” as the sole hashtag, and 23.6% of tweets (n = 3298) used a second hashtag. To better understand the hashtags accompanying #digitalinclusion, a word cloud was created based on all 14,000 tweets (Figure 1). The most frequent co-occurring hashtag was #digitalequity, mentioned in 1027 tweets (7.3% of the total sample). Other top hashtags included #accessibility (802 tweets, 5.7%), #digitaldivide (715 tweets, 5.1%), #digitalskills (528 tweets, 3.8%), and #digitaltransformation (514 tweets, 3.7%). 1420 (10.1%) of the tweets included at least one emoji, and 713 (5.1%) included two emojis. Based on the subsample of 1000 consecutive tweets in the qualitative data, 101 different emojis were found. The total number of emojis used in the whole dataset was not calculated.[Fig fig2-20552076231211277]

#### Media and links

The different kinds of media types were: photos, animated graphic interchanged format (GIF), and video. Of the total sample, 49.1% (n = 6879) had some media embedded in the tweet. However, in calculations of the subsample, including only tweets with embedded media, photos were the most common media type, accounting for 89.4% (6149/6879). Similarly, 10.6% (728/6879) had a GIF embedded or a video in the tweet; the two types were equally distributed. The whole dataset was also searched for tweets that had an URL embedded but had no media attached to the tweet; these amounted to 6811 (49.2%). Because many of the links were shortened URLs (bit.ly, or equivalents), it was not possible to calculate the origins of the URLs.

#### Origin of users and their tweets

There were 3931 unique user accounts, and these users posted between one and 368 tweets. 104 different clients were used to tweet. The most frequently used was “Twitter Web App” (37.7%), followed by “Twitter for iPhone” (13.5%), “Twitter for Android” (10.1%), “Hootsuite Inc.” (9.4%), and “TweetDeck” (7.4%). The most active user had 368 tweets, alone publishing 2.6% of all 14,000 tweets. The second most active user posted 306 tweets (2.2% of the total) and the third most active user posted 276 tweets (2.0% of the total). A large number of the user accounts were registered several years ago, for example 1637 (41.6%) were registered between 2009–2012; for a detailed overview see Figure 1.

The number of followers ranged between zero and 4,978,238, with a mean of 12,018 and a median of 1101. The user with the highest number of followers was “Intel” followed by “World Economic Forum,” “Nokia,” Digital India,” “Verizon,” “airtel India,” TECH4ALL,” “FCC” (US Federal Communication Commission). User account activity was also measured by the number of total tweets. The least active user accounts only had one tweet, while the most active one, had 976,190 tweets in total. The mean value was 10,058 and the median value 2883. About one fifth of the unique users, 21.2% (n = 2970), had a website attached to their user biography account. Regarding disclosure of contact information, only nine accounts disclosed their phone number, and 116 revealed their email address. Owing to the possibility to hide one's location as a Twitter user, 1968 tweets could not be located to a geographical position. Thus, the remaining 12032 tweets had disclosed a position. The analysis showed that users who tweet about digital inclusion are from different continents: USA, Europe, Africa, and Asia. A map is available at https://www.google.com/maps/d/edit?mid=15Px64DHFE7Ff5xUMVBl0zHLdD6T31_A&ll=58.84941425591368%2C17.20469076850651&z=7 (Supplement 1). The map shows the geographic location of the sample. The densest area of tweets is the west coast of the US and the UK and Ireland. More detailed calculations were not made on this variable.

### Qualitative results

The qualitative thematic analysis resulted in four themes.

#### Criticizing the discourse and warning about digital exclusion

This theme covered tweets that had taken initiatives by writing that digital inclusion is not enough, thus warning about the consequences: digital exclusion. One tweet serves as an illustrative example: “It's unacceptable to hear [author: *blinded name*] from @DigiPovAllience mention 33% of young people have no access to home broadband at @HACThousing's #DigitalInclusion Network Launch this afternoon. We are meeting with other organizations to discuss what we can do to help. #DigitalExclusion” (Tweet #547). Similarly, another tweet warns that many older people are excluded as they are not active on the web: “40% of those aged 75 + are not online. Many more not confident online. Yet #OlderPeople often trying to access services from #banking to #health where there are no offline options or online support. #exclusion #DigitalExclusion #DigitalInclusion https://t.co/UiSM0pzxea” (Tweet:#843). Lack of relevant infrastructures for students in rural areas was also criticized: “Off-campus students lack equitable access to #broadband, researchers say https://t.co/t9E6bzvvgl via @HigherEdDive #DigitalInclusion #DigitalEquity #highereducation https://t.co/Nxj6q6k6zT” (Tweet: #723). On an aggregative level, the critique was that access to affordable broadband should be a human right: “FOUR million people are missing out on cheap broadband, [author: *blinded name*] has warned. https://t.co/oh9xsClf1w #Digital #DigitalInclusion #DigitalExclusion #ConnectionsForAll #Broadband #SocialTariffs” (Tweet: #408). Thus, the potential risks of being digitally excluded were stressed in one tweet: “A lack of digital skills and access can have a huge negative impact on a person's life, leading to poorer health outcomes and a lower life expectancy, increased loneliness and social isolation, less access to jobs and education. https://t.co/XqWxGuq8W3 #digitalinclusion https://t.co/fAa6HpfnQz” (Tweet: #506). Similarly, a few tweets were interpreted as including connotations to cynicism: “Out with the old, in with the new” It's not that easy when making sure our #leadership team is digital- and change ready! Just bringing in new people can’t be the answer. We have to enable people being open for change and embrace the new. #digitalinclusion #transformation https://t.co/W6THWMUiH3” (Tweet: #952).

#### Upvoting and broadcasting initiatives and efforts to improve digital inclusion

Twitter was used as a platform to broadcast incentivizers’ own efforts to increase digital inclusion. “.@[author: *blinded name*]is donating up to 400 Chromebooks and some of the mobile phones used by the Census Field officers this year to voluntary and charitable organizations across Scotland. For more info and to apply head to 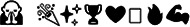 https://t.co/c9IknAbl4s #DigitalInclusion https://t.co/IaESU1Ccse” (Tweet: #446). In this case, one Twitter account wanted to communicate a contribution of ICT to individuals who were socially disadvantaged. In one tweet, a service invited users with digital problems to seek help: “Having problems with your device? Pop in and see us at our drop in clinics or call 01606 305 007 for more information. Tue 15 Nov, Tue 29 Nov and Tue 13 Dec between 10am-1pm Castle Community Centre, Barbers Lane, Northwich CW8 1DT #ITsupport #Cheshire #digitalinclusion https://t.co/OMxxhLE2W6” (Tweet: #163). Another tweet upvoted a partnership intended to achieve digital equity and digital inclusion through broadband technology in rural areas of New York State: “Great! Where else can you do this, @Verizon? Verizon Enters into New York Public-Private Partnership for Rural #Broadband https://t.co/9g5Sm4eXUP via @telecompetitor #DigitalInclusion #DigitalEquity #ruralbroadband” (Tweet: #715). Signs of personal engagement in the endeavor for digital inclusion were also found in the data: “En route to Glasgow. Did I mention that I really love trains? I am on my way to DigiFest. #Digifest2022 is one of the most instructive and well received events of the Digital Calendar #Digifest2022 #DigitalInclusion #digitaltechnology #digitalhealthcare #DigitalEvolution #SCVO https://t.co/4uRMYBH25S” (Tweet: #692). Upvoting was not restricted to omnipotent tweets, as several tweets upvoted others’ initiatives: “Dear friends, thank you for generously supporting our founder's candidacy for the 2022 @WomenTechNet global ‘Women in ICT’ Award 2022 by sharing the post below on social media! #digitalequity #digitalinclusion #girlsintech #womenintech https://t.co/kSZe51PYjl” (Tweet: #57).

#### Challenging others to improve and provide facilitators and barriers

Tweets were interpreted as challenging others to act and to push others to improve their efforts to incentivize digital inclusion: “Did you know you can help your parents, grandparents, friends, siblings and others learn the basics of internet with our FREE online resources? Oh yes, all of our lessons online for you to use… FREE. Click here to learn now https://t.co/kVCMhm2n6v #DigiKick #DigitalInclusion https://t.co/liQktj4iTV” (Tweet: #889). One account challenges others to improve digitalization and digital inclusion by appealing to their competitive instinct: “Where does your city rank? (Buffalo NY is 84/100): The stark disparity across internet access in the US https://t.co/kN1hXoDTXD via @CityMonitorAI #DigitalInclusion #DigitalEquity #Broadband https://t.co/nUyNd0FVog” (Tweet: #720) and “Closing the #digitaldivide is a marathon and a sprint for cities https://t.co/jk3QSE1U5e via @Cities_Today #DigitalInclusion #DigitalEquity https://t.co/BLHrpEgtOP” (Tweet: #779). Other “psychological manipulations” were also seen in tweets, for example “Digital inclusion should consider both your employees and customers to ensure they have access to digital and the skills and training required. Red (*sic*) more here - https://t.co/OiKQwb1xeG #digitalinclusion #DigitalDivide #DigitalEquality” (Tweet: #789).

#### Fueling the debate

Tweets also dealt with digital inclusion in general. These tweets were interpreted as being more neutral in their appearance, because they did not include any imperative or explicit attempt to affect others. Instead, they used Twitter to broadcast good examples and good outcomes of facilitators and barriers for digital inclusion: “At #ConnectingScotland we get some amazing stories of all the ways a device and internet connection have changed someone's life! #DigitalInclusion https://t.co/tJFLxvhTER” (Tweet: #471). Digital inclusion targeted different groups of individuals. One tweet concerned the advantages of recording university lectures: “The benefits of recorded lectures - from a student's perspective. #InclusiveUCC #UniversityCollegeCork #UDL #DigitalInclusion #OneSmallChange https://t.co/DkK6NZo1 × 3” (Tweet: #872). Another tweet fueled the debate by presenting the voice of older adults: “The use of digital #technology continues to grow, however, devices continue to lack inclusivity of the rights and needs of #olderadults. What role does nursing play in #digitalinclusion? Read more at https://t.co/b1w64gnh4t #AWorld4AllAges https://t.co/o9XGzUF6R5” (Tweet: #803), and yet another tweet focused on women in low- and middle-income countries: “What can be done to accelerate #DigitalInclusion for women across low- and middle-income countries? This @GSMA report contains actionable recommendations for policymakers across 4 action areas identified by the @UNBBCom https://t.co/tLnDOnEvJP” (Tweet: #190).

## Discussion

The present study set out to explore and describe how Twitter users share information about digital inclusion. The study is based on 14,000 tweets posted on Twitter during a period of about 15 months. A subsample of 1000 tweets published during a five-week period in 2022 were analyzed using qualitative thematic analysis. The results showed that tweets about digital inclusion are driven by four different facilitators and barriers: tweets that warns against the risks of digital exclusion; tweets that promote actions to increase digital inclusion; tweets that call for others to take action to improve digitalization; and tweets that are neutral but fuel the debate by being active. Regarding the quantitative analysis, it was found that retweeting was rare, as only about 2% of tweets were retweeted ten times or more. Scholars have previously investigated the motivations and factors underlying retweeting behavior.^46,47^ Majmundar and colleagues^47^ developed the *Why We Retweet Scale,* in which four factors are pronounced: to s*how approval*, to argue, *to gain attention and to entertain. Comparison of the findings with those from other studies explicitly confirms the aspect of “attention.” Moreover, the results showed that almost half of the tweets had an embedded URL in which readers can read more about an initiative.*

In the early days of the internet, there was widespread belief that adopting digital technologies would automatically lead to greater social and economic development. However, it became clear that this was not the case, and that many individuals and communities were left behind, both in developed and developing countries.^48,49^ This led to a growing recognition of the need for digital inclusion efforts, which aim to ensure that everyone has the opportunity to participate in the digital economy and to reap the benefits of digital technologies. The results presented here demonstrate that several facilitators and barriers are already in place, for example, donations of Chromebooks and offering technical support as well as the importance of reminding others that everyone is not digitally included. However, as a counterpart to the inducements, one category of tweets shared the features of criticizing and warning about digital exclusion. These tweets were interpreted as voicing the need for others to take action to support digital inclusion in the future, and they shared the same vital spirit illustrated in the Swedish children's song “Idas sommarvisa,”^50^ which begins “Du ska inte tro det blir sommar, ifall inte nån sätter fart” (in English approximately; “You shouldn't count on it being summer unless someone gets things moving”). Such calls for others to take action also accords with observations in earlier studies of social media activism.^29,30,36^ The results demonstrate that some Twitter accounts have many followers, for example, “Intel,” “World Economic Forum,” “Nokia,” Digital India,” “Verizon,” “airtel India,” TECH4ALL,” “FCC” (US Federal Communication Commission). Even though these accounts were not at the top in tweets about digital inclusion, they still have an impact when posting about digital inclusion, as they have a position as opinion leaders.

### Digital inclusion – a matter of health equity

Social services and healthcare have seen tremendous progress in digitalization during recent years. However, the risk of becoming digitally excluded in a digitalized society has raised concerns in previous studies^51–53^ Thus, scholars have stressed the need for digital inclusion to ensure that digital equity is achieved for all citizens and that the services align with the SDGs in Agenda 2030.^4,19,49,52^ The World Bank publishes a worldwide index (with a 0-1 scale) for digital adoption on three dimensions: people, government and business. Only 22 countries have a score higher than 0.8. However, digital adoption is not necessarily equivalent to digital inclusion. For example, Sweden and the Netherlands are ranked with a score above 0.8, but inquiries have shown that some people are digitally excluded, which makes exclusion an even bigger issue for some as a consequence of the broad digitalization of public services and healthcare.^54,55^ Thus, being on the outskirts of digitalization in a digitalized society entails the risk of being excluded from contact with healthcare and social services.^56–58^ Taking all of this into consideration, the critical voices found in the present results serve as an important counterpoint to only seeing the bright side of digitalization – which has been termed the oxymoron of digitalization.^59^ Hence, the results of LDA modeling suggest that discussions centered on grassroots digital inclusion efforts and sustainable technology resonated strongly with the Twitter audience. This highlights an opportunity for policymakers and organizations to further support and promote small-scale, community-driven initiatives advancing digital inclusion. By empowering local programs through funding and resources, broader digital equity and sustainable development goals can be realized through community engagement.

The findings reveal that Twitter users are actively engaged in posting on the topic of digital inclusion. They show that the topic engrosses people on a global scale, people from both developed and developing countries. This also accords with earlier studies discussed in the background literature review of small-scale initiatives and large-scale programs.^34,35^ It is concluded that digital inclusion is needed to establish equity in living conditions for individuals and societies and that actions are needed that are in line with the fundamentals of SDGs in Agenda 2030.^4,16^ When individuals are “digitally included” they can access health information and services and, thereby, thrive. Digital technologies can provide individuals with access to a wide range of health information and services, including medical advice, health education, and remote monitoring of chronic conditions.^60,61^ This can be particularly beneficial for individuals who live in rural or underserved areas, or for those who have difficulty accessing traditional healthcare services. Digital health technologies can make it easier for individuals to access health information and services at their own convenience, rather than having to schedule appointments or travel long distances to see a healthcare provider,^62^ or to address health issues that public healthcare does not deal with.^63^ Moreover, increased efficiency and cost savings might well be associated with digital inclusion, as digital technologies can help to streamline delivery of health services, making it easier for healthcare providers to manage and coordinate care.^64,65^ The insights gained from the present study have been that providing individuals and societies access to ICT and digital literacy education is of vital importance in promoting digital inclusion, thus, stressing the overall intention *to leave no one behind*. There are still many unanswered questions concerning how social media fuel digital inclusion. Future investigations should use a larger sample and address in more detail research questions concerning what motivates the various initiative to engage in social media.

### Limitations

The trustworthiness of the qualitative component remains robust, adhering to the criteria of credibility, transferability, dependability, and confirmability as outlined by Lincoln and Guba^43^ and reiterated by Nowell et al.^44^ specifically for thematic analyses. However, the study has several limitations. While the specific focus on #digitalinclusion allowed for an in-depth analysis of tweets directly discussing digital inclusion, it is possible relevant tweets were missed by not including hashtags representing associated concepts (for example #digitalequity). The narrow focus on a single hashtag limits the breadth of the analysis but was an intentional choice to avoid conceptual conflation. It is worth noting that, in comparison to other qualitative inquiries on Twitter data, the sample size in this study is not an outlier. For instance, Michaela et al.^66^ analyzed 1357 tweets posted over approximately one month to investigate district communication during the pandemic. Nisch^67^ examined public opinion on Finland joining NATO by using a subset of 500 random tweets, which were manually reviewed and analyzed. Lee et al.^68^ conducted a qualitative content analysis of only 700 tweets from health-related users, posted over a three-day period. These studies demonstrate that smaller sample sizes are not uncommon in qualitative Twitter research. However, given the specific aim of the current study the sample size of 1000 tweets could still be considered a limitation for the qualitative thematic analysis component. The themes identified may not be fully representative, particularly since the qualitative data were derived from a sample spanning over about one month. Additionally, the larger sample size of 14,000 tweets from a period of about 15 months serves as a contrasting point, highlighting the main weakness of the study in terms of thematic depth is the main weakness of the study.

The LDA modeling provides unique quantitative insights into topics and perspectives that spur high engagement on social media. The methodology demonstrates how statistical modeling can uncover patterns in large text datasets. Other interesting findings to emerge were found in the qualitative data. Thus, from the perspective of qualitative epistemology, sample size is not as important as in nomothetic epistemologies.^42^ For this reason, the present article does not make claims regarding external validity, rather qualitative method scholars would argue for the findings’ transferability.^43^ Although it is possible to hide the location of a Twitter account, only 14.5% chose to hide their geo-position. Notably, the geographic position of tweets is partly skewed because some locations are very detailed, while others are equally non-specific: “Africa,” “13 major cities across India,” or simply “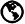.” A major challenge for the social media platform Twitter is the number of non-human accounts or bots. It is difficult to accurately estimate the number of bot accounts on Twitter, because bots can be designed to mimic human behavior, making them difficult to detect. Twitter has taken steps to identify and remove bot accounts, but it is likely that some bot accounts still exist on the platform. Some studies have estimated that a significant portion of Twitter accounts are bots, but the exact percentage is uncertain^69–71^; thus, it cannot be ruled out that some tweets in the present sample were produced by bots. An estimation made nearly ten years ago indicated that about 8.5% of user accounts were bots.^72^ However, the sample originated from 3931 unique accounts, of which 41.6% were registered between 2009 and 2012; thus, it is unlikely that a bot active so long ago would be posting about digital inclusion. One possibility would have been to exclude non-verified accounts; however, this would have reduced the sample to only 1285 tweets. Because a high percentage of accounts were registered over ten years ago and are still active on the topic of digital inclusion, it is argued that such old accounts are held by real Twitter users.

## Conclusions

The present study concludes that digital inclusion is a topic that engages Twitter users globally. Tweets that were associated with community and local initiatives and sustainable development had the highest engagement in terms of number of retweets and likes. The interpretation of the studied tweets is that digital inclusion is of vital importance to achieving equity in living conditions and to improving access to health information and health services, among other services. The tweets indicate that lack of broadband connection and low digital literacy run the risk of undermining the imperative of “leaving no one behind.” Initiatives have been taken to increase digital inclusion, but more efforts are needed. The benefits of being digitally included can be seen on both an individual and a societal level.

## Supplemental Material

sj-xlsx-1-dhj-10.1177_20552076231211277 - Supplemental material for Digital inclusion: A mixed-method study of user behavior and content on Twitter Click here for additional data file. Supplemental material, sj-xlsx-1-dhj-10.1177_20552076231211277 for Digital inclusion: A mixed-method study of user behavior and content on Twitter by Martin Salzmann-Erikson in DIGITAL HEALTH

sj-docx-2-dhj-10.1177_20552076231211277 - Supplemental material for Digital inclusion: A mixed-method study of user behavior and content on Twitter Click here for additional data file. Supplemental material, sj-docx-2-dhj-10.1177_20552076231211277 for Digital inclusion: A mixed-method study of user behavior and content on Twitter by Martin Salzmann-Erikson in DIGITAL HEALTH
