# Supplementary material for: Digital inclusion: A mixed-method study of user behavior and content on Twitter
Source: Digit Health. 2023 Nov 1;9:20552076231211277. doi: 10.1177/20552076231211277 (PMC10621295; doi:10.1177/20552076231211277)
Supplement: sj-docx-2-dhj-10.1177_20552076231211277 - Supplemental material for Digital inclusion: A mixed-method study of user behavior and content on Twitter [file sj-docx-2-dhj-10.1177_20552076231211277.docx]

Supplementary 1: The map shows the geographic location of the sample.

<https://www.google.com/maps/d/edit?mid=15Px64DHFE7Ff5xUMVBl0zHLdD6T31_A&usp=sharing>
